# The Role of Heat Shock Protein 90B1 in Patients with Polycystic Ovary Syndrome

**DOI:** 10.1371/journal.pone.0152837

**Published:** 2016-04-05

**Authors:** Li Li, Hui Mo, Jing Zhang, Yongxian Zhou, Xiuhong Peng, Xiping Luo

**Affiliations:** 1 Department of Obstetrics and Gynecology, Guangdong Women and Children Hospital, Guangzhou, Guangdong, China; 2 Laboratory of Chinese Medicine Quality Research, Macau University of Science and Technology, Macau, China; 3 Guangzhou Family Planning Specialty Hospital, Guangzhou, Guangdong, China; University of Geneva, SWITZERLAND

## Abstract

Polycystic ovary syndrome (PCOS) is a heterogenetic disorder in women that is characterized by arrested follicular growth and anovulatory infertility. The altered protein expression levels in the ovarian tissues reflect the molecular defects in folliculogenesis. To identify aberrant protein expression in PCOS, we analyzed protein expression profiles in the ovarian tissues of patients with PCOS. We identified a total of 18 protein spots that were differentially expressed in PCOS compared with healthy ovarian samples. A total of 13 proteins were upregulated and 5 proteins were downregulated. The expression levels of heat shock protein 90B1 (HSP90B1) and calcium signaling activator calmodulin 1 (CALM1) were increased by at least two-fold. The expression levels of HSP90B1 and CALM1 were positively associated with ovarian cell survival and negatively associated with caspase-3 activation and apoptosis. Knock-down of HSP90B1 with siRNA attenuated ovarian cell survival and increased apoptosis. In contrast, ovarian cell survival was improved and cell apoptosis was decreased in cells over-expressing HSP90B1. These results demonstrated the pivotal role of HSP90B1 in the proliferation and survival of ovarian cells, suggesting a critical role for HSP90B1 in the pathogenesis of PCOS. We also observed a downregulation of anti-inflammatory activity-related annexin A6 (ANXA6) and tropomyosin 2 (TPM2) compared with the normal controls, which could affect cell division and folliculogenesis in PCOS. This is the first study to identify novel altered gene expression in the ovarian tissues of patients with PCOS. These findings may have significant implications for future diagnostic and treatment strategies for PCOS using molecular interventions.

## Introduction

Polycystic ovary syndrome (PCOS) is a heterogeneous disorder of the reproductive system in women that is characterized by follicular growth and anovulatory infertility. Genetic background is considered as one of the factors underlying PCOS development [1]. Surgical dissection of polycystic ovary is a major therapeutic option for the late stage of PCOS. However, the underlying molecular mechanism is not well understood. Recent clinical studies have indicated that PCOS is associated with endocrine and metabolic syndromes. Affected women are at risk of developing diabetes with impaired fasting glycemia and glucose tolerance [2–4]. In addition, PCOS is associated with stress based on recent studies in animal models demonstrating the critical role of chronic cold stress [5,6] and testosterone [2] in PCOS development, manifested as large cystic follicles and blocked ovulation following exposure to the environment. Recent studies have also shown changes in the expression of multiple genes in the ovarian tissues of patients with PCOS. Thus, the identification and modulation of expression of the altered genes provide a therapeutic approach to the treatment of PCOS. Recently, Salilew-Wondim et al. reported that a total of 573 genes were upregulated and 430 genes were downregulated in the ovaries of a patient with PCOS [1]. The most downregulated genes were associated with the biosynthesis and metabolism of steroids, cholesterol and lipids, whereas the most upregulated genes were associated with cell differentiation, proliferation, cell adhesion and blood vessel development [1]. Ambekar et al. reported similar results, in which a total of 186 genes were dysregulated in patients with PCOS. Among the identified 770 genes, all were relevant to follicular development, cell survival and apoptosis, such as proliferation of cell nuclear antigen (PCNA), amphiregulin, heparan sulfate proteoglycan 2, tumor necrosis factor, alpha-induced protein 6, plasminogen and lymphatic vessel endothelial hyaluronan receptor 1 [7]. However, to date, whether and how these dysregulated genes contribute to the pathogenesis of PCOS has remained elusive.

Heat shock proteins (HSPs) are a family of stress-inducible proteins, the expression and activity of which are highly regulated by the extracellular microenvironment. HSPs are coupled with 100 client proteins to modulate downstream target gene expression and activity by affecting protein folding and degradation. Their expression levels significantly affect cell survival, apoptosis and proliferation, and circadian clock gene expression under physiological and pathological conditions [8]. Multiple HSP90 isoforms have been identified, including cytosolic HSP90AA1, HSP90AB1 and endoplasmic reticulum (ER)-localized HSP90B1[9]. The HSP90 isoforms share common biological functions in cell survival and proliferation. It has been reported that high expression levels of HSP90B1, also known as gp96 and Grp94, are correlated with cancer cell survival and epithelial ovarian cancer [10]. Suppression of HSP90B1 expression can greatly reduce cell survival and biological function. For example, reduced expression of HSP90B1 induced slower oocyte growth and greatly decreased the thickness of the zona pellucida [11]. In HSP90B1-deficient macrophages in mice, an elevated bacterial burden and increased inflammation are observed. These debilitating effects are caused by the disrupted trafficking and function of Toll-like receptors (TLRs) and integrins in the affected cells [12]. Thus, the suppression of HSP expression and activity by HSP inhibitors has potential applicability in anti-tumor therapy and the prevention of excess inflammation-induced tissue damage [13–18]. Although the involvement of HSPB1 activity in cancer growth has been well described in recent years, the role of HSP90B1 in the pathogenesis of PCOS has not been well investigated. Recent studies in animal models indicated that adrenocorticotropic hormone or cold stress-induced PCOS were associated with increases in HSP90B1, glucocorticoid receptor and androgen receptor expression in ovarian tissues, indicating the possible function of the upregulated genes in the pathogenesis of PCOS [19].

In addition, HSP90B1 protein is involved in the suppression of cell apoptosis and autophagy, as demonstrated by a recent study showing that HSP90B1 inhibitor promoted cell apoptosis and autophagy [15]. HSP90B1 inhibitor is able to degrade HSP90B1 client proteins and increase cytochrome c release, activation of caspase-3, and expression of rapamycin complex 1 (mTOR) and LC-3 [15,20–22]. Because HSP90B1 is a dominant isoform in the HSP90 family and is critically involved in disease development [23,24], we investigated its protein expression profile by proteomic analysis using ovarian tissues from 10 patients with PCOS, and we defined the role of HSP90B1 in the pathogenesis of PCOS.

## Methods

### Patients

A total of 10 patients with ovarian polycystic syndrome (PCOS) were included in this study. The diagnosis of PCOS was based on the manifestation of chronic oligo-anovulation and/or hyperandrogenism and/or ultrasonography of ovarian morphology according to the National Institutes of Health and the European Society for Human Reproduction and Embryology/American Society of Reproductive Medicine [25]. Three grams of tissue were harvested from PCOS patients who had undergone laparoscopic ovarian wedge resection. A total of 10 healthy ovarian tissue samples were also obtained as controls from the contralateral ovary of patients with unilateral ovarian tumors, reserving 3 grams of normal tissue that were collected when the patients received section biopsies. The PCOS group and the control group were matched for age and weight. This study was approved by the Ethics and Research Committee of the Guangdong Women and Children’s Hospital, Guangzhou, China, and all patients signed written informed consent forms.

### Cell culture

Granulosa cells were isolated from follicular fluid, which was obtained from individual follicles of PCOS patients at the time of laparoscopy. Cells were cultured in DMEM supplemented with 10% fetal bovine serum (Gibco, Invitrogen, USA), and they were maintained in a humidified incubator at 37°C with 5% CO_2._

### Immunohistochemistry

Ovarian tissues were embedded in OCT medium for sectioning. Sections (7 μm) were fixed with 4% paraformaldehyde and then permeabilized with 1% Triton X-100. After washing twice with PBS, the sections were incubated with antibodies against S phase related proliferating cell nuclear antigen (PCNA) (Santa Cruz, CA, dilution 1:500), HSP90B1 (CST, dilution 1:200), CALM1, ANXA6, or TPM2 (ABcam, dilution 1:200) for 2 hours. After washing twice with PBS, the sections were incubated with secondary antibodies conjugated to HRP for 1 hour. Finally, the sections were washed 3 times and developed by addition of DAB substrate. Positively stained cells were visualized under a light contrast microscope. Images were obtained using a Nikon Eclipse, TE 2000-U fluorescence microscope.

### Western blot analysis

Five grams of ovarian tissues were homogenized in 200 μl RIPA lysis buffer (Promega, USA) to prepare the protein extracts. After centrifugation for 20 minutes, the protein samples were assayed using BCA (Pierce). Proteins (20 μg) were loaded into a 10% Tris-SDS PAGE gel. The separated proteins were transferred onto a PVDF membrane for protein detection. The protein on the blots was incubated with primary antibodies against PCNA, cleaved caspase-3 (CST, dilution 1:750), HSP90B1, CALM1, ANXA6 and TPM2 (Abcam, dilution 1:500) for 2 hours. Antibody against GAPDH served as an internal control. After two washes, the blots were incubated with HRP-conjugated secondary antibodies (R&D) for 1 hour. Finally, the blots were developed with ECL (Pierce) and scanned using an ImageQuant LAS 4000 biomolecular imager equipped with a CCD camera system (Springer Lab). Standard curves were generated during the quantitative analysis via the serial dilution of one protein extract sample and resolution by PAGE. Target gene protein and the internal control protein GAPDH were detected after incubation with the antibodies. Quantitative analysis of target gene protein was performed in the linear range of the standard curve. Protein extract samples with a band density outside the linear curve were diluted and analyzed by Western blot a second time. Data are presented as the mean ratio of the density of the target protein to GAPDH.

### Flow cytometry analysis

Two grams of ovarian tissue was digested with 100 mg/ml collagenase A for 2 hours, and the single cell suspension was stained with propidium iodide (PI) and Annexin V conjugated to FITC. After a 10-minute incubation on ice, the stained cells were washed twice with PBS containing 3% FBS and fixed with 4% paraformaldehyde for 10 min for FACS analysis. Flow cytometry analysis was performed using a BD FACSCalibur flow cytometer (BD Biosciences, San Jose, CA). All data were analyzed using FlowJo software, version 8.8.4 (Tree Star Inc.).

### HSP90B1 siRNA transfection

For the knockdown experiments, small interfering RNA targeting HSP90B1 (5’-GAAGAAGCAUCUGAUUACCTT-3’) were designed to target HSP90B1 mRNA (NM_003299) sequences. As a nonspecific control, a NC siRNA with random sequences was designed as follows: 5’-AGUUCAACGAGUAUCAGCATT-3’. All siRNAs were synthesized by Shanghai GenePharma (Shanghai, China). Small interfering RNAs (200 pmol per well) were added using Lipofectamine 2000 for transfection (Invitrogen, California, USA) according to the manufacturer's protocol. 48 hours after transfection, cell lysates were harvested and analysed using Western blot.

### Difference in-gel electrophoresis (DIGE)

Protein samples (50 μg) were labeled with 400 pmol Cydye (GE Healthcare) according to the manufacturer’s instructions. The samples were labeled with Cy3 and Cy5. The internal standard controls were pooled samples labeled with Cy2. Labeled samples were applied to a 24-cm immobilized pH gradient gel strip ranging from pH 3 to pH 10 for the first dimension of isoelectrophoresis. After isoelectric focusing, the strips were subjected to the second dimension of electrophoresis in DALT Six (GE Healthcare) for 12 hours. Finally, the gels were scanned using a Typhoon 9400 imager. The data were analyzed using DeCyder 2D software V6.5 (GE Healthcare). The protein expression levels were calculated as the density ratio of the individual sample to the internal standard control. In addition, a pooled sample without dye labeling was stained with Coomassie blue G-250 after separation on a preparative gel. The spots on the preparative gel that matched with spots on the dye-labeled gel were automatically selected and picked up using an Ettan Spot Picker for further analysis (GE healthcare).

### Protein identification

The selected spots were destained with 50% acetonitrile and 100 mM NH4HCO3 for 10 min. After gel dehydration and concentration, the proteins in the spots were extracted and digested for the peptide preps. The samples of each spot were analyzed by MALDI-TOF/TOF mass spectrometry (Applied Biosystems). The analyzed protein was identified using the MAS-COT search engine against the Swiss-Pro non-redundant sequence database (Version 2.1 Matrix Science). The search profiles were created using GPS explore software (Version 3.6.2, Applied science).

### Statistical analysis

The Student t-test was used for the statistical analysis. All data were compared to the normal ovarian controls.

## Results

### PCNA was increased, but caspase-3 cleavage was decreased, in ovarian tissues from patients with PCOS

**Fig 1:**
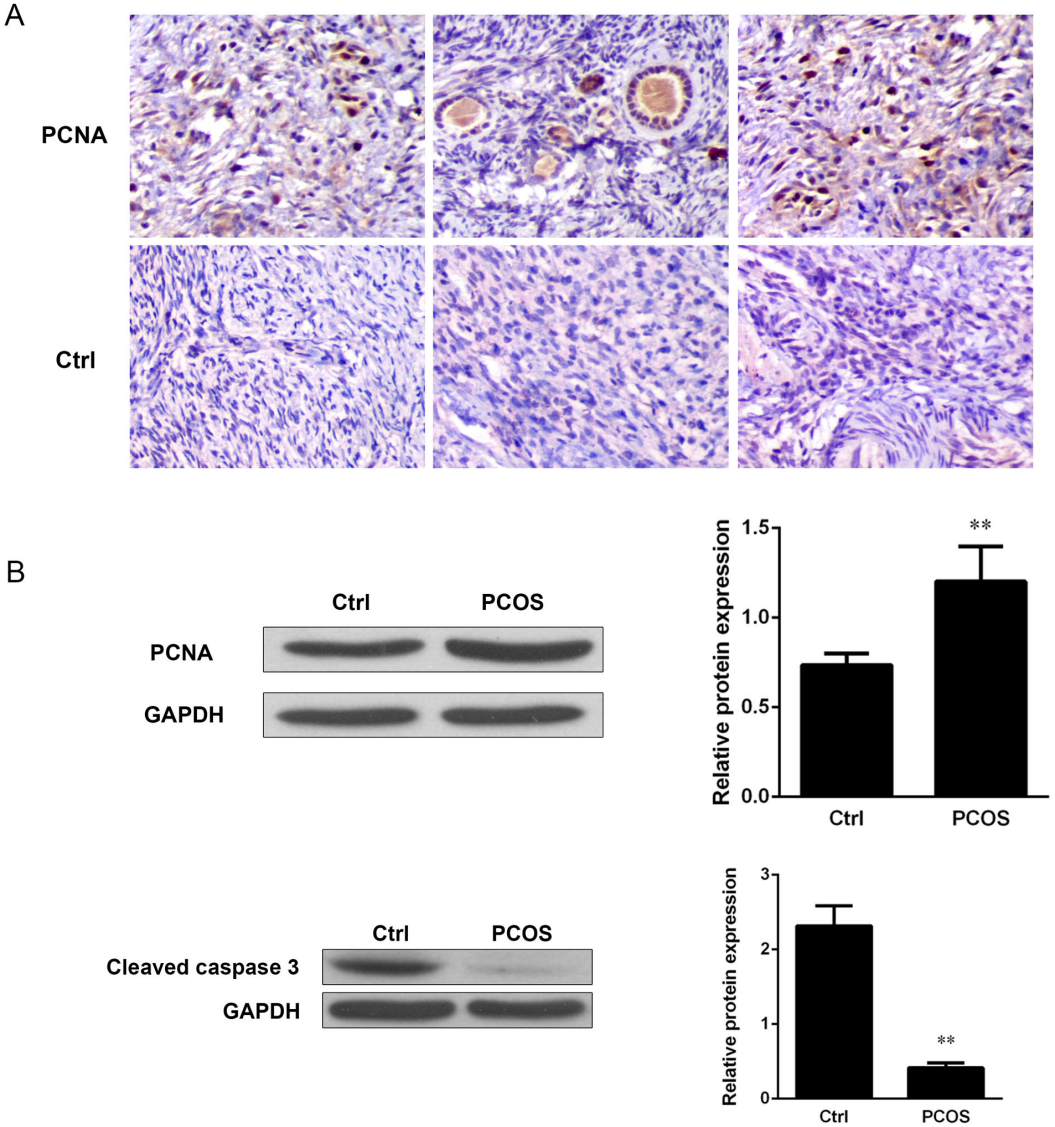
PCNA and caspase-3 cleavage were increased in the ovaries of patients with polycystic ovarian syndrome (PCOS). (A) S phase related proliferating cell nuclear antigen (PCNA) was analyzed by immunohistochemistry. The sections of ovarian samples from patients with PCOS and healthy ovarian tissues were stained with an antibody against PCNS (1:200 dilution) for 2 hours, followed by incubation with a secondary antibody. The stained sections were developed with DAB substrates and visualized under a light contrast microscope (magnification 20 X). One representative result is shown. The expression levels of PCNA (B, left panel) and cleaved caspase-3 (C, upper panel) were analyzed by Western blot analysis. Immunoblots were incubated with anti-PCNA antibody (dilution 1:500) and anti-cleaved caspase-3 antibody (dilution 1:750). GAPDH antibody (dilution 1:5000) was used as an internal control. One representative result is shown. The expression levels of PCNA (B, right panel) and cleaved caspase-3 (C, lower panel) were analyzed quantitatively by densitometric analysis based on the results of the Western blot analysis. Data represent the mean ratio of the densitometric density of detected proteins to GAPDH ± standard error, n = 10.

We hypothesized that PCOS might be associated with increased cell proliferation and apoptosis. To test this hypothesis, we analyzed cell proliferation by measuring the expression of S phase related proliferating cell nuclear antigen (PCNA) and the cleavage of caspase-3 by immunostaining and Western blot analysis. Our results revealed elevated positive staining for PCNA protein in the ovarian tissues from patients with PCOS as compared with the normal controls (Fig 1A). The elevated PCNA expression was further confirmed by Western blot analysis and quantitatively analyzed by densitometric analysis. We observed an increase in PCNA expression by at least 1.6-fold compared with the controls (*P*<0.01) (Fig 1B). To define the association between cell proliferation and cell apoptosis, caspase-3 activation was measured by Western blot analysis. We observed a reduction of caspase-3 cleavage in the ovarian tissues from patients with PCOS compared with those from controls, and further quantification by densitometric analysis revealed an 80% decrease in caspase-3 activation (*P*<0.01) (Fig 1C). Thus, we concluded that there was an increase in ovarian cell proliferation but a suppression of cell apoptosis in the ovarian tissues of patients with PCOS.

### Protein analysis by 2D-DIGE analysis

**Fig 2:**
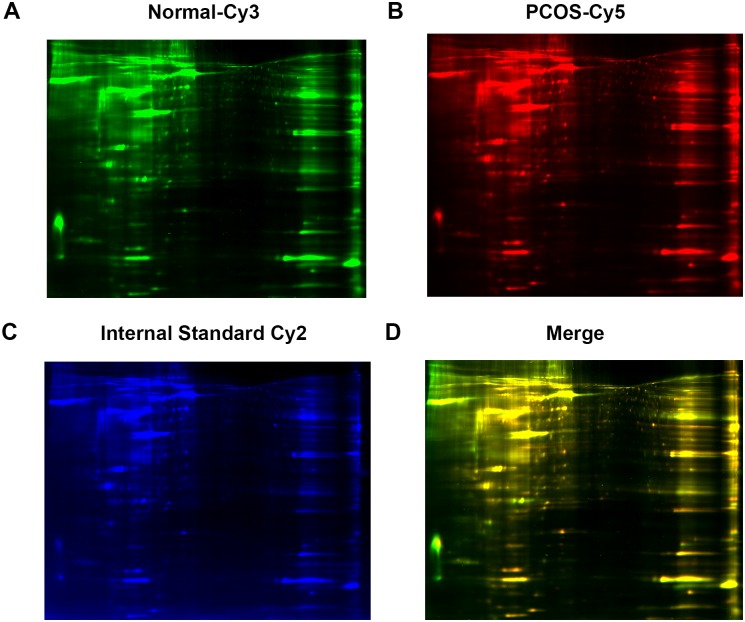
Protein analysis by 2D-DIGE analysis. Ovarian tissues from patients with PCOS and healthy controls were analyzed for differential protein expression by 2D-DIGE. A total of 10 samples were analyzed in each group. Individual samples were labeled with Cy3 (A) and Cy5 (B). Pooled samples were labeled with Cy-2 as an internal control. The three labeled samples were then mixed and loaded into a 2D-PAGE gel for two dimension separation. The labeled proteins were visualized and scanned for imaging. An overlay of three dye-scanned images was obtained (D). One representative result from 10 individual samples is shown.

To analyze the protein expression profiles in the ovarian tissues, 2D-DIGE isoelectrophoresis and subsequent MALDI-TOF/TOF mass spectrometer analysis were performed using individual samples from patients with PCOS (n = 10). As shown in Table 1, a total of 18 protein spots were identified with a statistically significant change in abundance in the samples from PCOS patients versus control patients (Fig 2). Thirteen proteins were upregulated including heat shock protein 90B1 (HSP90B1) and calcium signaling activator calmodulin 1 (CALM1), and 5 proteins were downregulated (Table 1) including anti-inflammatory activity-related annexin A6 (ANXA6) and beta-tropomyosin 2 (TPM2). All identified proteins are relevant to cell metabolism, mobility, proliferation, adhesion and survival, among other processes.

**Table 1:** Ovarian proteins with significant differences in abundance between normal control and PCOS samples.

Gene name	SwissProt ID	PI	MW (KD)	PCOS/control
**CALM1**	P62158	4.09	16.8	5.09
**SET**	Q01105	4.23	33.5	2.19
**YWHAE**	P62258	4.63	29.3	1.94
**HSP90B1**	P14625	4.76	92.7	1.84
**CALR**	P27797	4.29	48.3	1.75
**TUBB**	P07437	4.78	50.1	1.72
**PTRF**	Q6NZI2	5.51	43.4	1.65
**C1QBP**	Q07021	4.74	31.7	1.63
**VIM**	P08670	5.06	53.7	1.63
**GANAB**	Q14697	5.74	107	1.53
**RBP1**	P09455	4.99	16.01	1.53
**PGRMC1**	O00264	4.56	21771.8	1.51
**SERPINC1**	P01008	6.32	53.03	1.5
**HSPA5**	P11021	5.07	72.4	-1.52
**LMNA**	P02545	6.57	74.4	-1.55
**TPM2**	P07951	4.66	32.9	-1.58
**ALB**	P02768	5.92	71.3	-1.63
**ANXA6**	P08133	5.42	76.2	-1.77

### HSP90B1 and CALM1 were increased, but ANXA6 and TPM2 were decreased, in PCOS tissues

**Fig 3:**
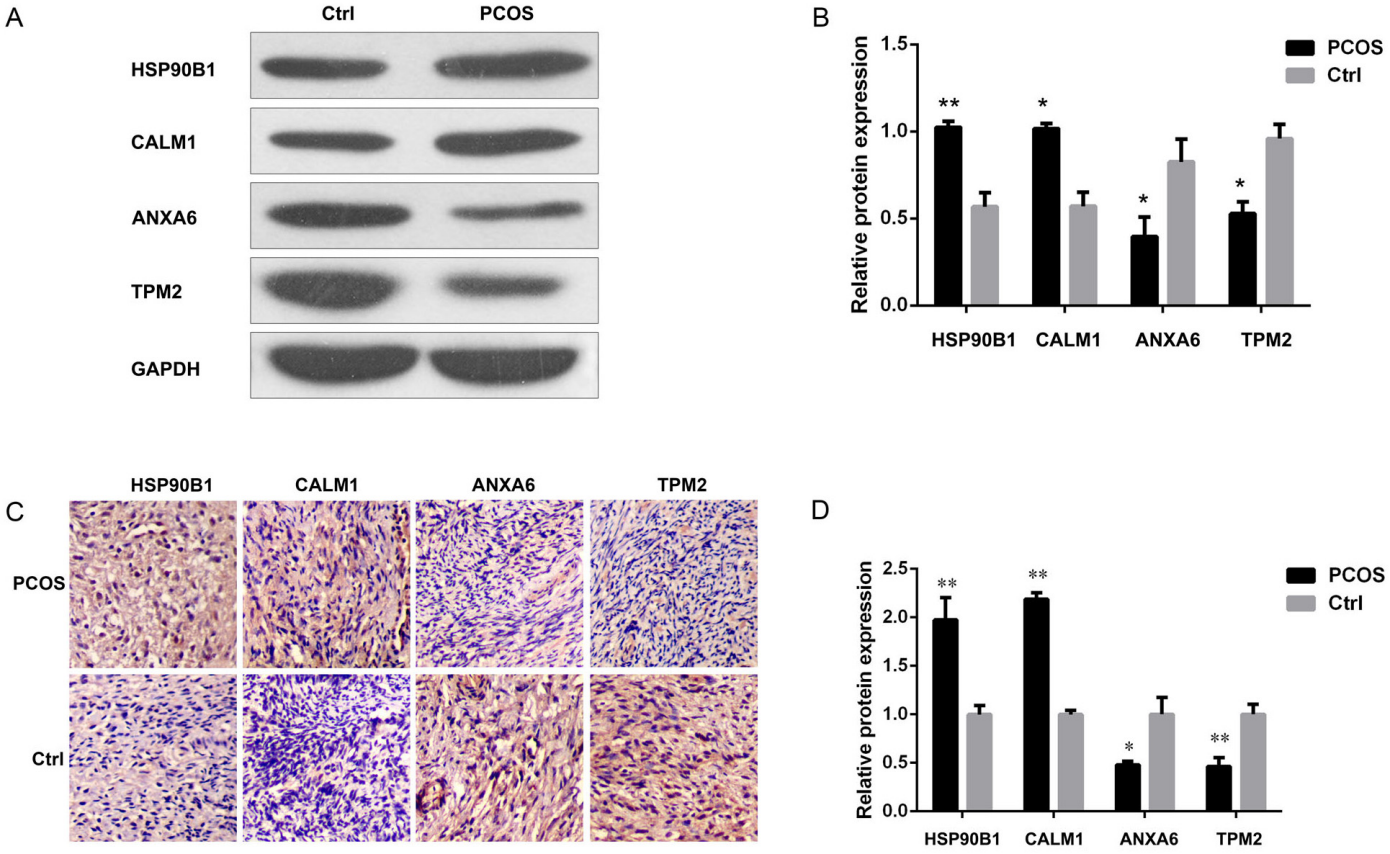
HSP90B1 and CALM1 were increased, but ANXA6 and TPM2 were decreased, in tissues from patients with PCOS. The expression of HSP90B1, CALM1, ANXA6 and TPM2 proteins from ovarian biopsies of control and PCOS were analyzed by Western blotting (A). One representative result is shown. GAPDH was used as the internal control. The protein expression were assessed quantitatively by densitometric analysis, (B) and the data are presented as the mean ratio of the target protein to GAPDH ± standard error. n = 5. (C) The expression levels of the identified proteins were analyzed by immunohistochemistry, and the stained sections were developed with DAB substrate. Protein expression was analyzed quantitatively by cell counting, and the data are presented as the mean ratio of the cell counts of positive cells to the nuclei ± standard error. n = 10.

To further confirm the expression of the identified proteins, we analyzed their expression levels by Western blot analysis. Protein extracts were obtained from the individual samples from two groups, and they were loaded into a 10% Tris-SDS PAGE gel for the detection of HSP90B1, CALM1, ANXA6 and TPM2. Consistent with the 2D-DIGE results, we observed elevated expression of HSP90B1 and CALM1, but suppressed expression of ANXA6 and TPM2, in the ovarian tissue samples from patients with PCOS compared to those from normal controls (Fig 3A). After normalization to the internal control, GAPDH, by densitometric analysis, we observed increases in both HSP90B1 and CALM1 by at least 2-fold (*P*<0.01 and *P*<0.05 respectively) but decreases in the expression levels of ANXA6 and TPM2 by 50% (*P*<0.05) (Fig 3B). The results were also further confirmed by immunostaining analysis, which revealed more abundant HSP90B1 and CALM1 and less abundant ANXA6 and TPM2 protein staining in the sections from patients with PCOS compared with those from normal controls (Fig 3C and 3D).

### There was more cell survival, but less cell apoptosis, in ovarian tissues from patients with PCOS

**Fig 4:**
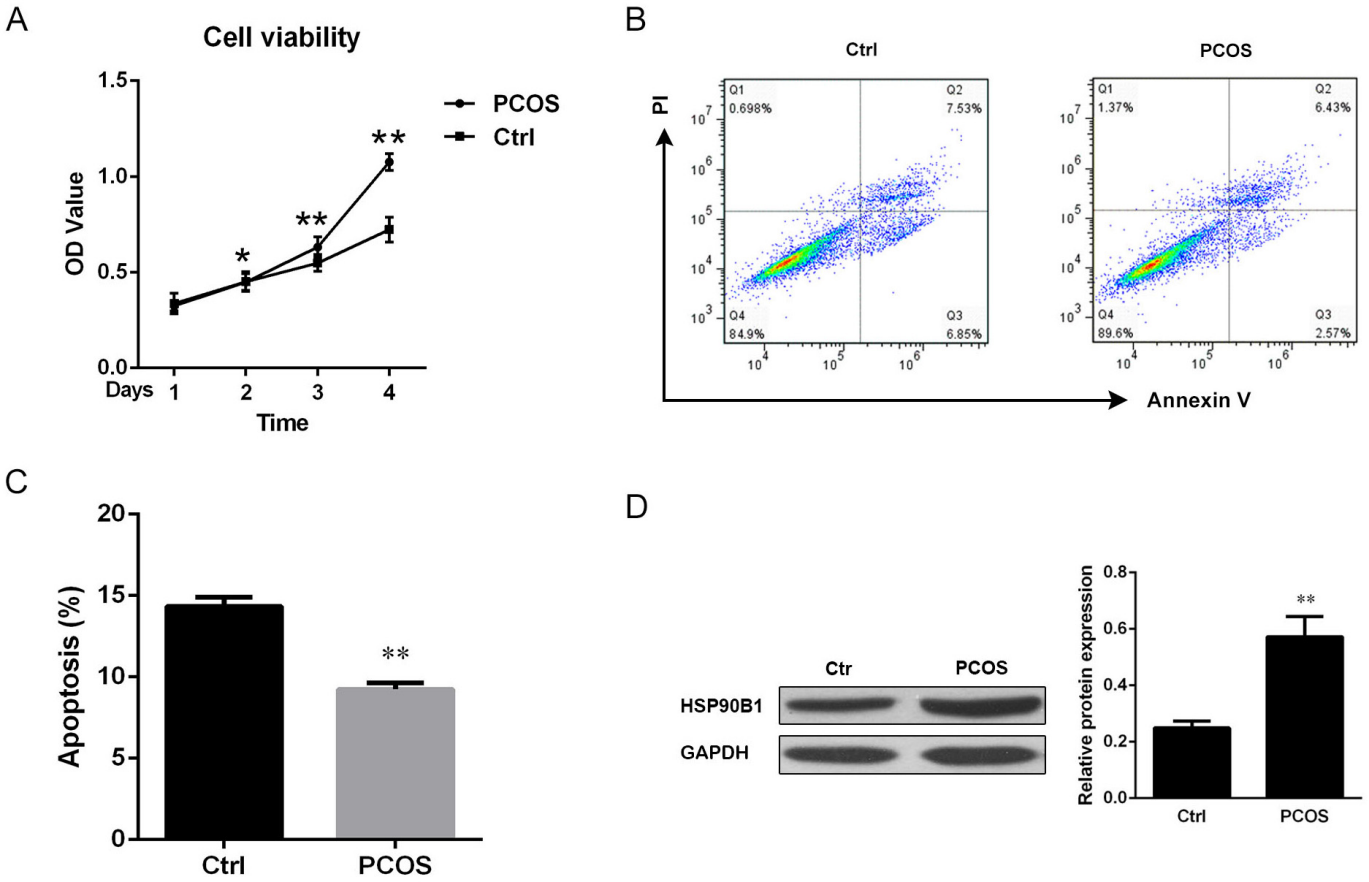
Cell apoptosis was decreased, but HSP90B1 was increased, in ovarian tissues from patients with PCOS. (A) Cell viability was analyzed by the MTT assay. Purified ovarian cells from Ctrl and PCOS were cultured in RPMI1640 medium for the indicated durations. Data are presented as the mean of the OD value from triplicate wells ± standard error. One of three independent results is shown. (B) The cell suspension from ovarian biopsy was stained with propidium iodide (PI) and Annexin V conjugated to FITC. The stained cells were analyzed by flow cytometry, and one representative result is shown. (C) Apoptotic cells (Annexin V+PI-) were quantitatively analyzed, and the data are presented as the mean percentage of apoptotic cells ± standard error, n = 10. (D) HSP90B1 protein expression levels were analyzed by Western blotting. The blots were incubated with anti-HSP90B1 antibody (dilution 1:1000), and one representative result is shown (left panel). The band density was quantitatively analyzed by densitometric analysis, and the data are presented as the mean ratio of the target protein relative to GAPDH ± standard error, n = 10 (right panel).

Because of role of HSP90B1 in cell survival and apoptosis, we next performed flow cytometry analysis to further identify the relationship between HSP90B1 expression levels and apoptosis. Single cell suspensions were cultured for the indicated number of days for cell viability analysis using the dimethylthiahiazo-di-phenytetrazoliumromide assay (MTT) (Life technology). A time-dependent increase in cell viability was observed in ovarian tissues from PCOS patients than from normal controls. Maximum differences in cell survival were observed at 4 days after culture (*P*<0.01) (Fig 4A). In addition, cell apoptosis was assessed by staining the cells with propidium iodide (PI) and Annexin V to distinguish viable, apoptotic and necrotic cells among the cultured ovarian cells. Because PI cannot permeate live and apoptotic cells, it only stains necrotic cells, whereas Annexin V can stain both apoptotic and necrotic cells. Our flow cytometry analysis revealed a decrease by more than 40% in the population of apoptotic cells (PI-Annexin V+) isolated from patients with PCOS compared with controls (*P*<0.01) (Fig 4B and 4C). The decreased population of apoptotic cells was associated with the HSP90B1 protein expression levels, with greater HSP90B1 protein levels but a reduced apoptotic cell population in the ovarian cells from patients with PCOS (*P*<0.01) (Fig 4D).

### Knock-down of HSP90B1 protein expression increased apoptosis in ovarian cells from patients with PCOS

**Fig 5:**
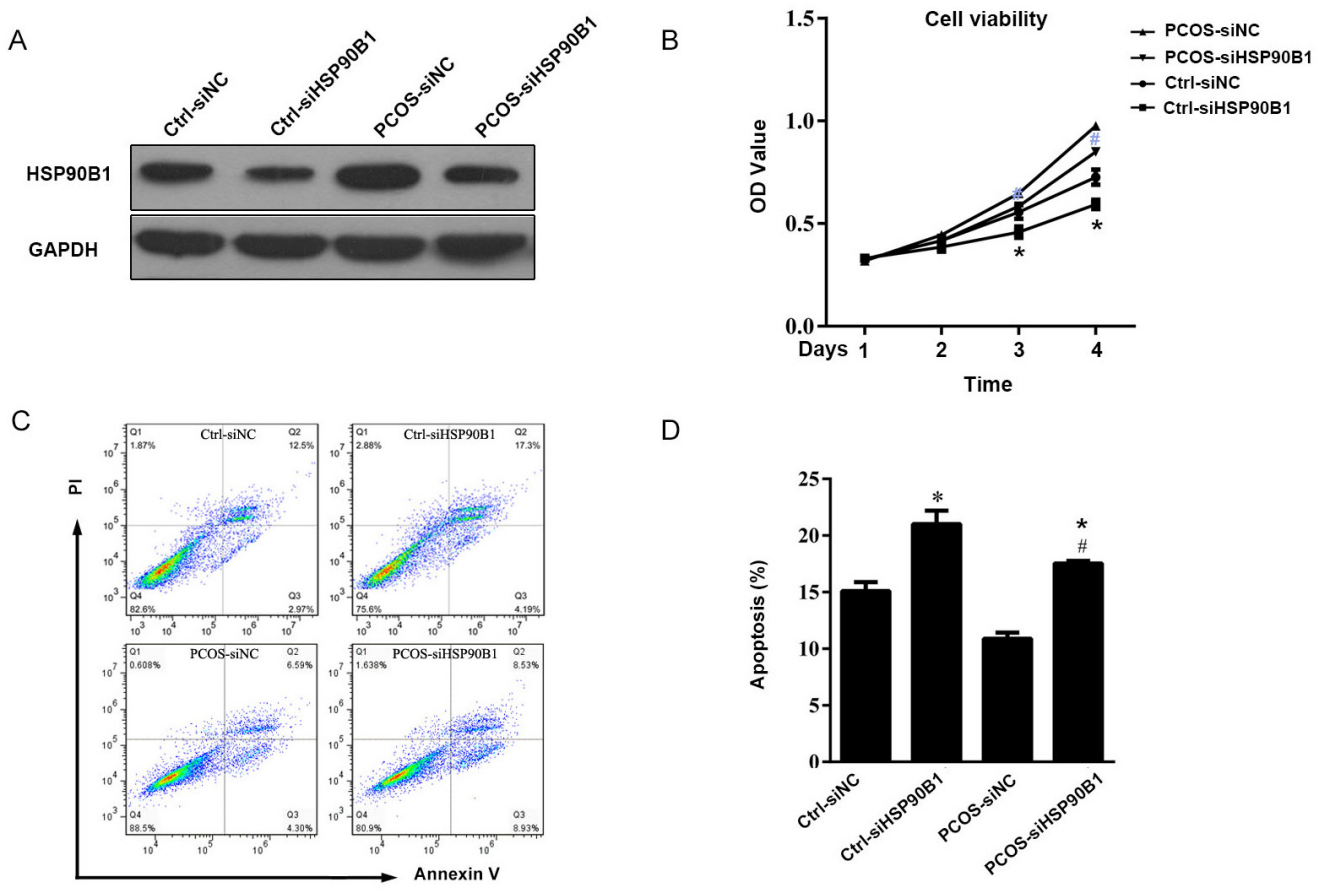
Knock-down of HSP90B1 protein expression increased apoptosis in ovarian cells. (A) The ovarian cells from patients with PCOS and controls were transfected with siRNA for 24 hours. Cells transfected with scramble siRNA served as control. At 48 hours post-transfection, the expression of HSP90B1 was analyzed by Western blot analysis using GAPDH antibody as as loading control. One representative result is shown. (B) The cells transfected with siRNA were used for the cell viability assay by MTT. Data are presented as the mean cell viability ± standard error, n = 10. (C) Cell apoptosis was analyzed by flow cytometry after staining with propidium iodide (PI) and Annexin V conjugated to FITC. Data are presented as dot plots, and one representative result is shown. (D) Apoptotic cells were analyzed quantitatively, and the data are presented as the mean percentage of apoptotic cells ± standard error, n = 10. (*P<0.05, compared with Ctrl-siNC; #*P*<0.05, compared with PCOS-siNC).

Because we observed a positive association between ovarian cell survival and HSP90B1 protein expression levels, we further analyzed the role of HSP90B1 in ovarian cell survival. Ovarian cells cultured in 6-well plate were transfected with 0.5 μg HSP90B1 siRNA encompassing exon 1 and exon 2 regions of the cDNA to knock-down HSP90B1 translation. The same amount of scramble siRNA was used as a negative control. At 48 hours after transfection, we observed a large decrease in the expression of HSP90B1 by Western blot analysis, as compared with the scramble siRNA-transfected controls. This result indicated that the knock-down of HSP90B1 expression was successful (Fig 5A). Further analysis indicated that the PCOS cells with knocked down HSP90B1 had a reduced viability than the PCOS cells transfected with scramble siRNA (*P*<0.01), and the cell viability of normal ovarian cells with knocked down HSP90B1 also had a lower than the cells transfected with scramble siRNA, supporting the importance of HSP90B1 in ovarian cell survival (Fig 5B). Consistently, there as a 1.8-fold increase in the proportion of PI-Annexin V+ apoptotic ovarian cells in PCOS and normal ovary cells transfected with HSP90B1 siRNA compared with the controls (*P* <0.01) (Fig 5C and 5D).

### Over-expression of HSP90B1 protein decreased apoptosis in ovarian cells from patients with PCOS

**Fig 6:**
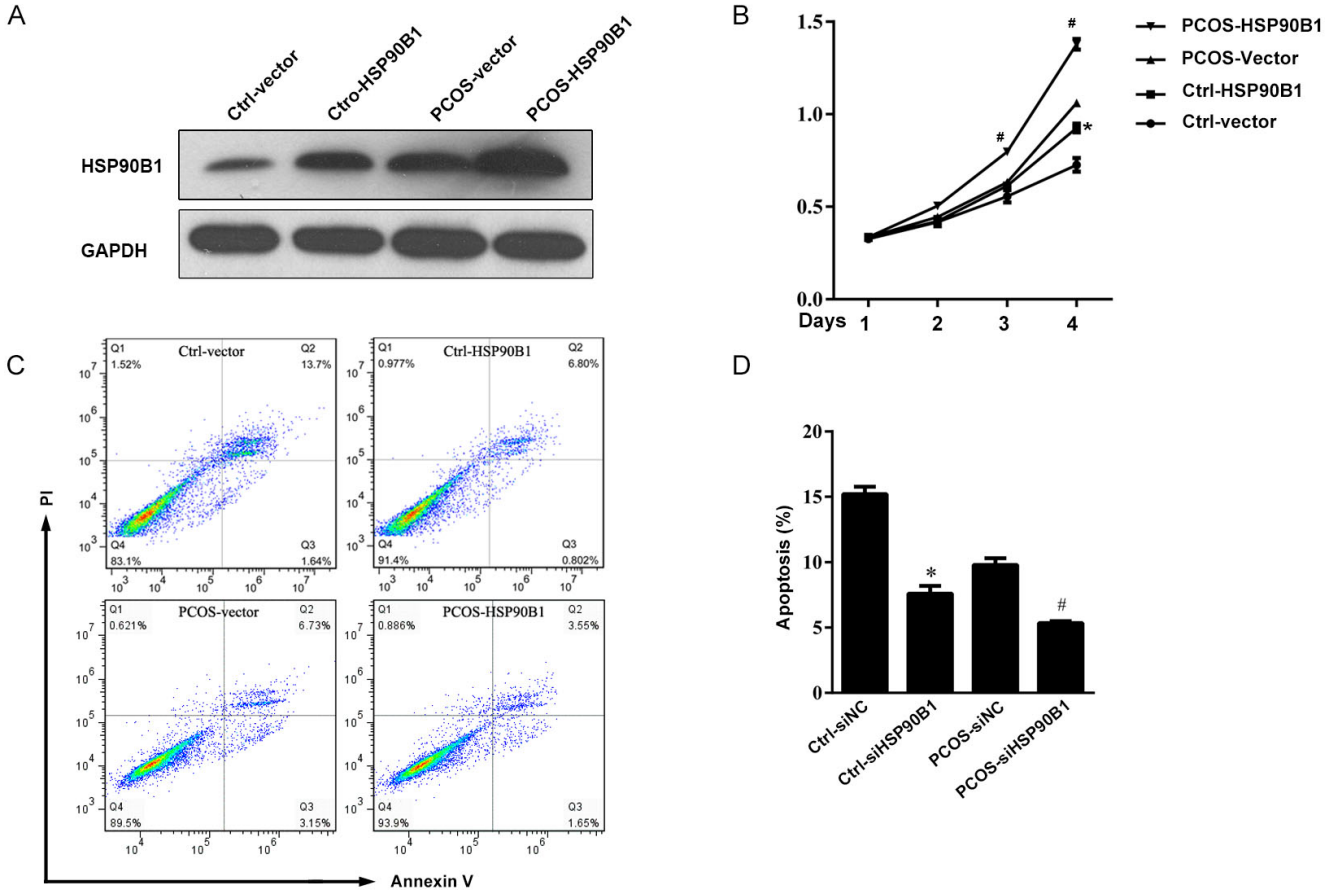
Over-expression of HSP90B1 protein decreased cell apoptosis in ovarian cells. (A) Ovarian cells were transfected for 24 hours with plasmid encoding HSP90B1; cells transfected with empty vector served as the control. At 48 hours post-transfection, the expression of HSP90B1 was analyzed by Western blot analysis, and GAPDH antibody was used as a loading control. One representative result is shown. (B) The cells transfected with plasmid encoding HSP90B1 were used for the cell viability assay using MTT. Data are presented as the mean cell viability ± standard error, n = 10. (C) The transfected cells were analyzed for apoptosis by flow cytometry after staining with propidium iodide (PI) and Annexin V. Data are presented as dot plots, and one representative result is shown. (D) Apoptotic cells were analyzed quantitatively, and the data are presented as the mean percentage of apoptotic cells ± standard error, n = 10. (*P<0.05, compared with Ctrl-vector; #*P*<0.05, compared with PCOS-Vector).

To confirm the role of HSP90B1 in ovarian cell survival and apoptosis, we transfected normal and PCOS ovarian cells with a plasmid encoding the HSP90B1 cDNA under the control of the CMV promoter. Cells transfected with empty vector were used as a control. At 48 hours post-transfection, HSP90B1 protein was over-expressed in ovarian cells from PCOS patients transfected with the HSP90B1 cDNA plasmid as compared with those transfected with empty vector. HSP90B1 protein was also over-expressed in normal ovarian cells compared with the control vector group (Fig 6A). Further analysis also indicated that ovarian cells over-expressing HSP90B1 had significantly improved survival in a time-dependent manner; maximum survival differences were observed after 4 days of culture (Fig 6B). In contrast, apoptosis of cultured PCOS cell was suppressed by at least 50% after transfection with the HSP90B1-expressing plasmid, as compared with the PCOS vector group. The apoptosis of normal ovarian cells was also reduced compared with the control vector group, supporting a critical role for HSP90B1 in cell survival and proliferation (*P*<0.01) (Fig 6C and 6D).

## Discussion

Anovulation, hyperandrogenism and polycystic ovaries are the main clinical manifestations of PCOS. Because the etiology of PCOS remains unclear, the identification of protein expression profiles in the ovarian tissues of patients with PCOS would benefit therapeutic strategies. Although genomic and epigenetic studies have revealed many candidate genes that are associated with PCOS phenotypes, additional genes remain to be investigated to achieve a better understanding of the pathogenesis of PCOS. In the present study, we identified a significant increase in the expression of HSP90B1 and CALM1, but a decrease in the expression of ANXA6 and TPM2 in the ovarian tissues from PCOS patients. Additional studies have indicated an important role of HSP90B1 in the pathogenesis of PCOS. Accompanying the increased HSP90B1 expression, we observed an increase in PCNA expression and a decrease in caspase-3 cleavage in the PCOS tissues, demonstrating an association between cell proliferation and apoptosis in the progression of PCOS. These results are consistent with the previous findings in which HSP90B1 was found to be highly involved in cancer cell growth and HSP90B1 inhibitors were able to reverse cancer cell growth and survival [15,21,26–30].

HSP90B1 is a stress-inducible chaperone protein. Its expression levels significantly affect cell proliferation and survival, cell cycle progression and apoptosis, as well as other features of malignant cells, such as invasion, tumor angiogenesis and metastasis. In addition to its known functions, our current study suggested the role for HSP90B1 in the promotion of cell proliferation in the pathogenesis of PCOS. Evidence has indicated that the dysfunction of granulosa cells affects folliculogenesis in patients with PCOS [31]. The increase in granulosa cell proliferation is associated with PCOS. In the ovaries of women with anovulatory PCOS, granulosa cells show higher proliferation levels compared with those with normal ovulation [32]. In the present study, increased expression of PCNA and decreased caspase-3 cleavage were observed in PCOS tissues, suggesting that there was an increase in ovarian granulosa cell proliferation in patients with PCOS. This is the first report to show that HSP90B1 is highly expressed in the ovarian cells of patients with PCOS and promotes the proliferation of granulosa cells. Our further in vitro studies have indicated that ovarian cells from PCOS patients possess increased caspase-3 activity and are more apoptotic after HSP90B1 knock-down, but this effect can be reversed by over-expressing HSP90B1. Thus, these observations suggest that HSP90B1 promotion of granulosa cell proliferation might be one mechanism contributing to the pathogenesis of PCOS. Modulation of HSP90B1 activity via molecular intervention may provide a novel strategy for the treatment of PCOS.

The molecular mechanisms underlying the role of HSP90B1 in ovarian cell function remain unknown. HSP90B1 is an important chaperone protein that interacts with multiple intracellular receptors and transcription factors, and it has a role in the stabilization of client proteins from protease degradation, such as AKT, IkappaB-alpha, glucocorticoid and progesterone receptors, among others [15,21,33]. These client proteins critically participate in cancer cell proliferation and survival. In addition, HSP90B1 is an important chaperone protein that is responsible for client protein stability and maturation after activation [34]. Growth factors and stress hormones are potent inducers of cancer cell proliferation and survival. These tumor-inducing effects are mediated by receptor driven downstream signaling pathways. HSP90B1 plays an important role in tumorigenesis by coupling with receptors to avoid receptor degradation prior to activation and maturation [35]. Thus, the inhibition of HSP90B1 activity would lead to stable tumorigenic receptors and attenuate tumorigenic activities associated with stress. Recent studies have indicated that HSP90B1 inhibitor is more effective than hormone and growth receptor inhibitors for anti-cancer growth. For example, HSP90B1 inhibitor can suppress the proliferation of non-small cell lung cancer (NSCLC) that is refractory to epidermal growth factor receptor (EGFR) inhibitor; downstream STAT3 and ERK1/2 activation was more effectively suppressed by HSP90B1 inhibitor [36,37]. Thus, further studies of the signaling pathways downstream of HSP90B1 will improve our understanding of the molecular mechanisms of HSP90B1 in PCOS tumorigenesis.

In addition, we observed an increase in the calcium signaling activator calmodulin 1 (CALM1) in the ovarian tissues from PCOS patients. CALM1 is linked to the differentiation of osteoblasts. Its expression can be down-regulated by dominant negative MCP-1, resulting in the suppression of human osteoclast differentiation [38,39]. Receptor activation by NF-kappaB ligand (RANKL) induces osteoclast differentiation through the activation of Ca2+/calmodulin-dependent protein kinases (CaMKs) [40]. However, whether the elevated CALM1 expression has an impact on ovarian cell proliferation and differentiation in PCOS remains unknown. Further investigations are needed to elucidate the importance of CALM1 in the pathogenesis of PCOS. In addition, we also observed a decrease in anti-inflammatory activity-related annexin A6 (ANXA6) and tropomyosin 2 (TPM2) in ovarian tissues from patients with PCOS. According to previous studies, ANXA6 is induced by antioxidant agents such as resveratrol [41]. Resveratrol-treated cells show increased expression of ANXA and autophagy-regulated genes such as gamma-aminobutyric acid A receptor-associated protein (GABARAP), microtubule-associated protein 1 light chain 3B (LC3B) and autophagy-related protein 3 (ATG3), and the radical scavenger activity-related metallothionein-1X (MT1X) genes [41]. Therefore, the suppression of ANXA6 expression in ovarian tissues of patients with PCOS potentially increases issue inflammation and cell survival, supporting its potential role in promoting ovarian cell proliferation and survival. Further studies will be performed to clarify the role of ANXA6 in the pathogenesis of PCOS.

Tropomyosin (TPM) comprises a family of actin binding proteins that are encoded by a group of highly conserved genes. These proteins are responsible for binding and stabilizing actin microfilaments. It has been reported that tropomyosin participates in tumorigenesis and ovarian tissue remodeling [42]. Over-expression of the TPM1 isoform can suppress tumor growth, such as squamous cell carcinoma proliferation in the esophagus [43,44]. Thus, TPM1 is a tumor growth suppressor [45]. We observed decreased expression of TPM2 in the present study, supporting the potential involvement of TPM2 in the pathogenesis of PCOS.

PCOS has a high level of etiological heterogeneity. In contrast to previous studies [46,47], we focused on the identification of altered proteomic biomarkers and the role of HSP90B1 in a Chinese population with PCOS. The clinical symptoms and the biological and molecular phenotypes varied on an individual basis. The development of proteomic analysis in recent years has become a very useful tool to study heterogeneity in PCOS, but the research approach is still on its infancy. Insenser et al. studied heterogeneity in PCOS using proteomic analysis, but the study was limited by a lack of standard criteria in terms of the sample collection and size. The statistical analysis tools differed from those used in the present study [47]. In addition, in a review article, Gupta et al. summarized the common metabolic signaling pathways in a heterogeneous population of patients with PCOS, indicating that the altered protein expression profiles were relevant to glucose metabolism, lipoprotein metabolism, cell proliferation, apoptosis, and insulin resistance [47]. In the present study, we identified for the first time novel proteomic biomarkers and investigated the role of the newly identified HSP90B1 in the pathogenesis of PCOS. These findings provide insights into a new role of HSP90B1 in the development of PCOS and reveal new diagnostic and therapeutic targets.

Overall, this is the first study to show that HSP90B1 and CALM1 are increased, but ANXA6 and TPM2 are decreased, in the ovarian tissues of patients with PCOS. The altered gene expression profile observed in patient with PCOS, particularly that of HSP90B1, has a significant impact on ovarian cell survival and proliferation in patients with PCOS. This study provides useful information for further investigations of PCOS pathogenesis and aids in the development of a novel strategy for the treatment of PCOS.

## Supporting Information

S1 Data Dataset used for the present findings. (XLSX)
